# Genome draft of the *Arabidopsis* relative *Pachycladon cheesemanii* reveals novel strategies to tolerate New Zealand’s high ultraviolet B radiation environment

**DOI:** 10.1186/s12864-019-6084-4

**Published:** 2019-11-12

**Authors:** Yanni Dong, Saurabh Gupta, Rixta Sievers, Jason J. Wargent, David Wheeler, Joanna Putterill, Richard Macknight, Tsanko Gechev, Bernd Mueller-Roeber, Paul P. Dijkwel

**Affiliations:** 1 School of Fundamental Sciences, Massey University, Tennent Drive, Palmerston North, 4410 New Zealand; 2 Department Molecular Biology, Institute of Biochemistry and Biology, University of Potsdam, Karl-Liebknecht-Straße 24-25, Haus 20, 14476 Potsdam, Germany; 3 School of Agriculture & Environment, Massey University, Palmerston North, 4442 New Zealand; 4 School of Biological Sciences, University of Auckland, Auckland, New Zealand; 5 Biochemistry Department, School of Biomedical Sciences, University of Otago, Dunedin, New Zealand; 6 Department of Plant Physiology and Molecular Biology, University of Plovdiv, 24 Tsar Assen str, 4000 Plovdiv, Bulgaria; 7 Center of Plant Systems Biology and Biotechnology (CPSBB), 139 Ruski Blvd, 4000 Plovdiv, Bulgaria; 8 Max Planck Institute of Molecular Plant Physiology, Am Mühlenberg 1, 14476 Potsdam, Germany

**Keywords:** Abiotic stress, *Arabidopsis*, Genome assembly, *Pachycladon*, UV-B tolerance

## Abstract

**Background:**

*Pachycladon cheesemanii* is a close relative of *Arabidopsis thaliana* and is an allotetraploid perennial herb which is widespread in the South Island of New Zealand. It grows at altitudes of up to 1000 m where it is subject to relatively high levels of ultraviolet (UV)-B radiation. To gain first insights into how *Pachycladon* copes with UV-B stress, we sequenced its genome and compared the UV-B tolerance of two *Pachycladon* accessions with those of two *A. thaliana* accessions from different altitudes.

**Results:**

A high-quality draft genome of *P. cheesemanii* was assembled with a high percentage of conserved single-copy plant orthologs. Synteny analysis with genomes from other species of the Brassicaceae family found a close phylogenetic relationship of *P. cheesemanii* with *Boechera stricta* from Brassicaceae lineage I. While UV-B radiation caused a greater growth reduction in the *A. thaliana* accessions than in the *P. cheesemanii* accessions, growth was not reduced in one *P. cheesemanii* accession. The homologues of *A. thaliana* UV-B radiation response genes were duplicated in *P. cheesemanii*, and an expression analysis of those genes indicated that the tolerance mechanism in *P. cheesemanii* appears to differ from that in *A. thaliana*.

**Conclusion:**

Although the *P. cheesemanii* genome shows close similarity with that of *A. thaliana*, it appears to have evolved novel strategies allowing the plant to tolerate relatively high UV-B radiation.

## Background

*Pachycladon* is an allopolyploid genus of the Brassicaceae family with eight perennial species endemic to the South Island of New Zealand and one species to Tasmania (Australia). These *Pachycladon* species are believed to have originated around 1–3.5 million years ago in New Zealand and are primarily distributed across the alpine regions of the South Island [1, 2]. *Pachycladon cheesemanii* is the most widespread of the *Pachycladon* species with a broad longitudinal distribution in New Zealand and a wide altitudinal range from 10 m to 1600 m above sea level [1].

*Pachycladon*’s allopolyploid genome (2*n* = 20) consists of two subgenomes which resulted from intra- or interspecific crossing [3]. Karyotype comparisons between extant *Pachycladon* species and the theoretical Ancestral Crucifer Karyotype showed that the chromosome structure had undergone multiple rearrangements prior to the allopolyploidy event taking place [4], and this has hampered efforts to trace back *Pachycladon*’s progenitors. Phylogenetic analysis of *Pachycladon* species based on five single-copy nuclear genes indicated that one of the genome copies was derived from the *Arabidopsis* lineage, while another was similar to both *Arabidopsis* and *Brassica* lineages [5]. However, a comparison of 547 homeologous gene pairs from *P. cheesemanii* and *P. fastigiatum* with the homologous genes from *Arabidopsis lyrata* and *Arabidopsis thaliana* found that no set of genes showed significantly different identity to *A. lyrata* and *A. thaliana* homologues, suggesting the two *Pachycladon* subgenomes are derived from the same lineage [6]. Data from analysis of the nuclear gene *CHALCONE SYNTHASE* (*CHS*) further supported the idea that both *Pachycladon* genome copies stem from the *Arabidopsis* lineage [7].

Polyploidization has been suggested to contribute to plants’ evolution and environmental adaptation under selection pressure [8–10]. Plants with polyploid genomes can benefit from functional diversification of redundant gene copies, with one gene copy retaining the original function, guaranteeing the plant’s regular growth and development, while the other can evolve to confer novel phenotypes, such as protection against challenging environmental conditions [11]. Thus, higher levels of UV radiation in New Zealand compared with locations in the Northern Hemisphere at similar latitudes may have contributed to the evolution of the *Pachycladon* species [12].

UV radiation is classified into three types, UV-A, UV-B and UV-C. While UV-C does not penetrate the atmosphere, some UV-B radiation reaches Earth’s surface, where it can damage important molecules like DNA. In order to acclimate to UV-B radiation, plants have developed multiple strategies, including reducing leaf area by curling of the leaves, inhibiting leaf and plant growth [13, 14] and increasing light reflection by inducing the production of a cuticular wax layer and the biosynthesis of light-absorbing secondary metabolites [15, 16]. Nevertheless, excess UV-B radiation can cause the development of hypersensitive response-like necrotic lesions and plant death [17–19].

UV-B radiation is perceived by the UVB-resistance 8 (UVR8) photoreceptor which was discovered by the UV-B hypersensitivity of the *uvr8* mutant [20]. The crystal structure of the UVR8 protein showed that its core domain consists of a covalently bound homodimer [21]. After UV-B radiation, this homodimer dissociates and monomeric UVR8 interacts with CONSTITUTIVE PHOTOMORPHOGENIC 1 (COP1) and transcription factors including ELONGATED HYPOCOTYL 5 (HY5) and HY5-HOMOLOG (HYH) to induce the expression of UV-B-responsive genes [22]. Induced genes included those that encode CHS, FLAVANONE 3-HYDROXYLASE (F3H) and FLAVONOL SYNTHASE 1 (FLS1), which are core enzymes involved in the biosynthesis of flavonoids [23] and are believed to function as a UV-absorbing sun screen [24]. Other induced genes include *PHOTOLYASE 1* (*PHR1*), which encodes protein phosphate starvation response 1, and *EARLY LIGHT-INDUCIBLE PROTEIN1* (*ELIP1*). ELIP1 plays a role in the interaction of UV-B-induced monomeric UVR8 with chromatin [25]. It was found that the UVR8-dependent pathway responds to a wide range of UV-B radiation (0.1–12 μmol m^− 2^ s^− 1^). Another less well-understood UV-response pathway was found that functions independently of UVR8. By treating *uvr8* mutants with relatively high UV-B radiation levels (1–12 μmol m^− 2^ s^− 1^), several genes induced by this pathway were identified [26].

Since *P. cheesemanii* survives in New Zealand's high UV-B radiation environments, this species may have evolved distinct UV-B-radiation response pathways. To learn how this species is able to cope in its unique environment, we first assembled a high-quality draft genome of *P. cheesemanii* and attempted to reveal the two highly similar subgenomes. The draft genome was used to identify *P. cheesemanii* candidate genes likely involved in UV-B radiation response pathways. However, interestingly, the UV-B-induced expression pattern of these genes differed from that observed in two *A. thaliana* accession with differing UV-B responses, suggesting that a distinct UV-B radiation response pathway has evolved in *P. cheesemanii* to enable adaptation to the high UV-B radiation environment in New Zealand.

## Results

### Genome assembly and assessment

We extracted *P. cheesemanii* Kingston genomic DNA for whole genome sequencing. The Illumina sequencing technology was used to obtain high coverage sequence reads to help us determine its ancestry and current gene-set. Paired-end and mate-pair libraries were sequenced and ~ 56 Gb of DNA sequence obtained. Raw reads (483,792,966 reads) were subsequently trimmed using the cutadapt algorithm that is present in the trim_galore package. Using k-mer analysis (Additional file 1) the genome size was estimated to encompass 596 Mb. Multiple aligners (Platanus and SOAPdenovo) with different k-mer lengths were used to generate genome assemblies. Subsequently, these assemblies were further evaluated using multiple metrics, and the best one (51-k-mer assembly) was selected based on the assembly size and N50 from Platanus (P.k51) (Additional file 2). The assemblies using SOAP resulted in a higher scaffold size compared to Platanus, but also a much higher number of gaps and lower percentage of complete single copy orthologues. Therefore, Platanus was used as the preferred genome assembler. The total assembly size using P.k51 was ~ 422 Mb and this represented 70.8% of the estimated genome size. The longest scaffold was 418 kb, while the number of scaffolds of length ≥ 500 and ≥ 1000 bases were 53,782 and 23,900, spanning ~ 300 Mb and ~ 280 Mb of assembly size, respectively. The N50 for the assembly (scaffolds ≥500 bp) was 24,761 bases (Table 1). This result indicated that the assembled genome draft was highly fragmented.

**Table 1:** Assembly statistics of the *P. cheesemanii* genome

	Platanus assembly
Total assembly size (bp)	422,560,840
Number of scaffolds (≥500 bp)	53,782
Longest scaffold (bp)	418,003
N50 (≥500 bp)	24,761
GC (%)	36.33
Number of Ns / 100 kb (bp)	749.01
Repeats (%)	42.96
Number of variants	434,467

A high amount of repetitive DNA in the genome could be one reason for the fragmented genome assembly [27]. Therefore, the repeat content in the genome draft was analyzed using different repeat identification tools, and it was estimated that ~ 43% of the total assembly size comprised repeat regions (Additional file 3 and Additional file 4). Among these, 15.96% were annotated as “retrotransposons”, 6.84% as “DNA transposons” and 19.89% as “unclassified repeats”.

BUSCO assessment revealed that 96.2% highly conserved plant orthologs were “complete”, 1.5% “fragmented” and 2.3% “missing”. Reads were mapped back to the assembly using Bowtie 2 to show 96.98% alignment (Table 2). The *P. cheesemanii* leaf transcriptome [6] was aligned against the assembled genome using PASA, and 97.94% of transcripts could be mapped to the genome (Table 2). A total of 47,821 protein coding genes were predicted using MAKER, with an average transcript size of 1544 bp and 4.42 exons per gene. With regard to non-coding RNAs, 115 rRNA, 707 tRNA and 209 miRNA genes were predicted. In addition, in a comparison of the alleles in *P. cheesemanii,* 434,467 SNPs and 123,778 SSRs were identified, highlighting the highly polymorphic information content of its genome (Additional file 5). Thus, the results showed a fragmented genome draft, which may be the result of the high number of repeat elements in non-coding regions or/and having two highly similar genomes to contend with. Nevertheless, the assembly of coding regions was deemed of high quality, based on BUSCO and PASA analyses.

**Table 2:** Assessment statistics of the *P. cheesemanii* genome

	Percentage (%)
Read alignment	96.98
Transcript alignment	97.94
BUSCO completeness	96.20

### Genome functional annotation

Each of the predicted genes was functionally annotated by using BLASTX against National Center for Biotechnology Information (NCBI) non-redundant protein [28] and Uniprot databases for green plants (Viridiplantae) (Table 3). About 84% of the predicted genes had a blast hit against either NCBI nr or Uniprot databases, or against both. Among these, 63% had a hit in the manually curated Swissprot database. Based on the BLASTX result against NCBI nr, the highest number of hits was with *Camelina sativa* (24.4%), followed by *Arabidopsis lyrata* (22.7%), *Arabidopsis thaliana* (19.0%) and *Capsella rubella* (17.3%), all belonging to the Brassicaceae family [29]. InterProScan identified protein signatures for 89.81% of the predicted proteins, and 2597 genes were classified as transcription factor (TF) encoding genes. Similar to *A. thaliana*, bHLH (239), MYB (212), ERF (211) and NAC (179) TFs comprised the largest TF families in *P. cheesemanii*. The predicted genes were used for classification into pathways using the KEGG database. Similar to other plant species, the terms “metabolic pathways” and “biosynthesis of secondary metabolites” were assigned to the largest numbers of the predicted genes in *P. cheesemanii* (2930 and 1594, respectively) (Additional file 6).

**Table 3:** Annotation statistics of the *P. cheesemanii* genome

Number of predicted genes	47,821
Average transcript length (bp)	1544.46
Average CDS length (bp)	941.27
Average number of exons per gene	4.42
Average exon length (bp)	212.92
Average intron length (bp)	176.32
Length of scaffolds (≥500 bp)	299,926,053
Length of scaffolds (≥1 kb)	279,782,042

### Synteny analysis of the *P. cheesemanii* genome draft within Brassicaceae species

**Fig. 1:**
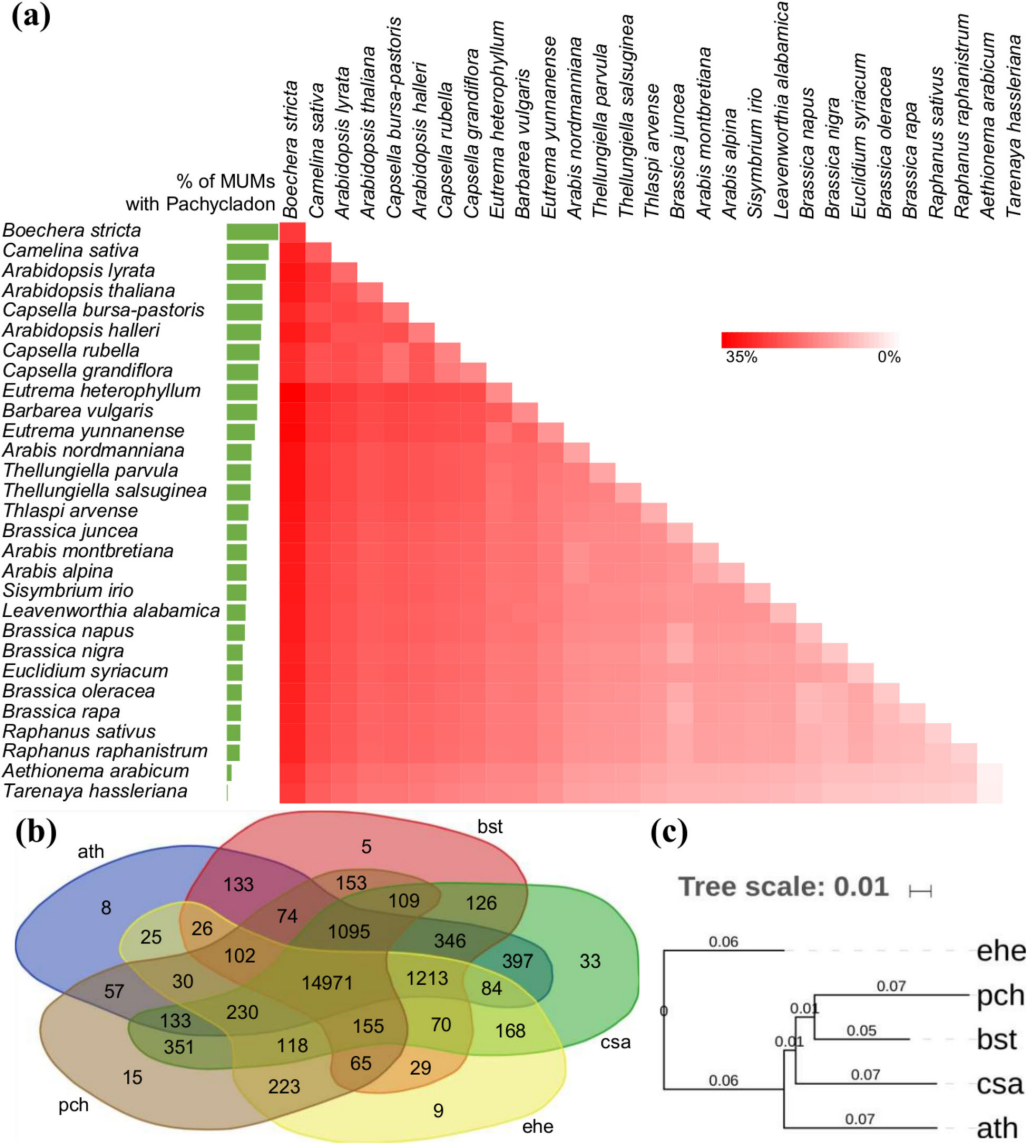
Prediction of the origin of the *P. cheesemanii* genome. **a** MUMmer alignment percentage (MUMs: Maximal Unique Matches) of *Pachycladon* against other sequenced Brassicaceae genomes. The numbers indicates cumulative percentage of MUMs for the respective pair of species against *P. cheesemanii*. **b** OrthoFinder output showing orthologous clusters between *P. cheesemanii* (pch), *A. thaliana* (ath), *B. stricta* (bst), *E. heterophyllum* (ehe) and *C. sativa* (csa). **c** Dendrogram of five species with high scores in MUMmer alignment. Numbers represent branch lengths

It has been reported that the two *Pachycladon* subgenomes originate from the hybridization of two species of the Brassicaceae family, one each from the *Arabidopsis* and *Brassica* lineages [5]. Here, the *P. cheesemanii* genome was aligned against all publicly available Brassicaceae genomes using MUMmer to perform synteny analysis. Of 28 available Brassicaceae genomes, seven each were from the Brassiceae and Camelineae tribes, four from the Eutremeae tribe, three from the Arabideae tribe, two from the Cardamineae tribe, and one each from the Thlaspideae, Sisymbrieae, Euclidieae, Boechereae, and Aethionemeae tribes (the tribes of Brassicaceae Lineage I: Camelineae, Cardamineae, and Boechereae; the tribes of Lineage II: Sisymbrieae and Brassiceae; the tribe of Lineage III: Euclidieae; the tribes of Expanded Lineage II (EII): Thlaspideae and Eutremeae; the tribe of the basal lineage: Aethionemeae; the unassigned tribe: Arabideae) [29]. *Tarenaya hassleriana* from the Cleomaceae family was selected as an outgroup [29]. Species with the highest alignment percentage (Maximal Unique Matches: MUMs) against the *P. cheesemanii* genome belong to Boechereae (29%), Camelineae (~ 20%) and Eutremeae (~ 15%). All pairwise combinations of the Brassicaceae genomes were used to estimate the cumulative alignment percentage with the *P. cheesemanii* genome to determine possible ancestral genomes of *Pachycladon*. The combination of *Boechera stricta* and *Eutrema heterophyllum* had the highest cumulative alignment with *P. cheesemanii* (37.35%) at the genome level (Fig. 1a).

From the species with the highest alignment percentage against the *P. cheesemanii* genome, three species from Brassicaceae Lineage I (*C. sativa*, *A. thaliana* and *B. stricta*, two from the Camelineae tribe, and one from the Boechereae tribe) and one from Lineage EII (*E. heterophyllum*, from Eutremeae tribe) [29] were selected for protein ortholog analysis. To identify orthologs, predicted proteins of all five species were blasted against each other in a pairwise manner for a total of 25 combinations. The BLAST searches were further processed using OrthoFinder to identify orthologs. A total of 182,585 genes (76%) were assigned to 20,553 orthogroups that included 14,971 orthogroups shared within the five species (Fig. 1b). For *P. cheesemanii*, 66.4% of the genes (31,749) were assigned to 87% (17,881) of the total orthogroups. Among these orthogroups, 15 novel orthogroups containing 72 genes were present in *P. cheesemanii*. Based on the orthogroups, a dendrogram of the five species was constructed (Fig. 1c). In accordance with the synteny analysis, *P. cheesemanii* showed the closest relationship with *B. stricta*, followed by *C. sativa* and *A. thaliana*. Beside the orthogroups that were shared by all species, *P. cheesemanii* shared the highest number of orthogroups with *C. sativa* (2191), followed by *B. stricta* (1753), *A. thaliana* (1721) and *E. heterophyllum* (923). Thus, the data suggests that *P. cheesemanii* has a closer phylogenetic relationship with species from Lineage I of the Brassicaceae family than to those of Lineage EII.

**Fig. 2:**
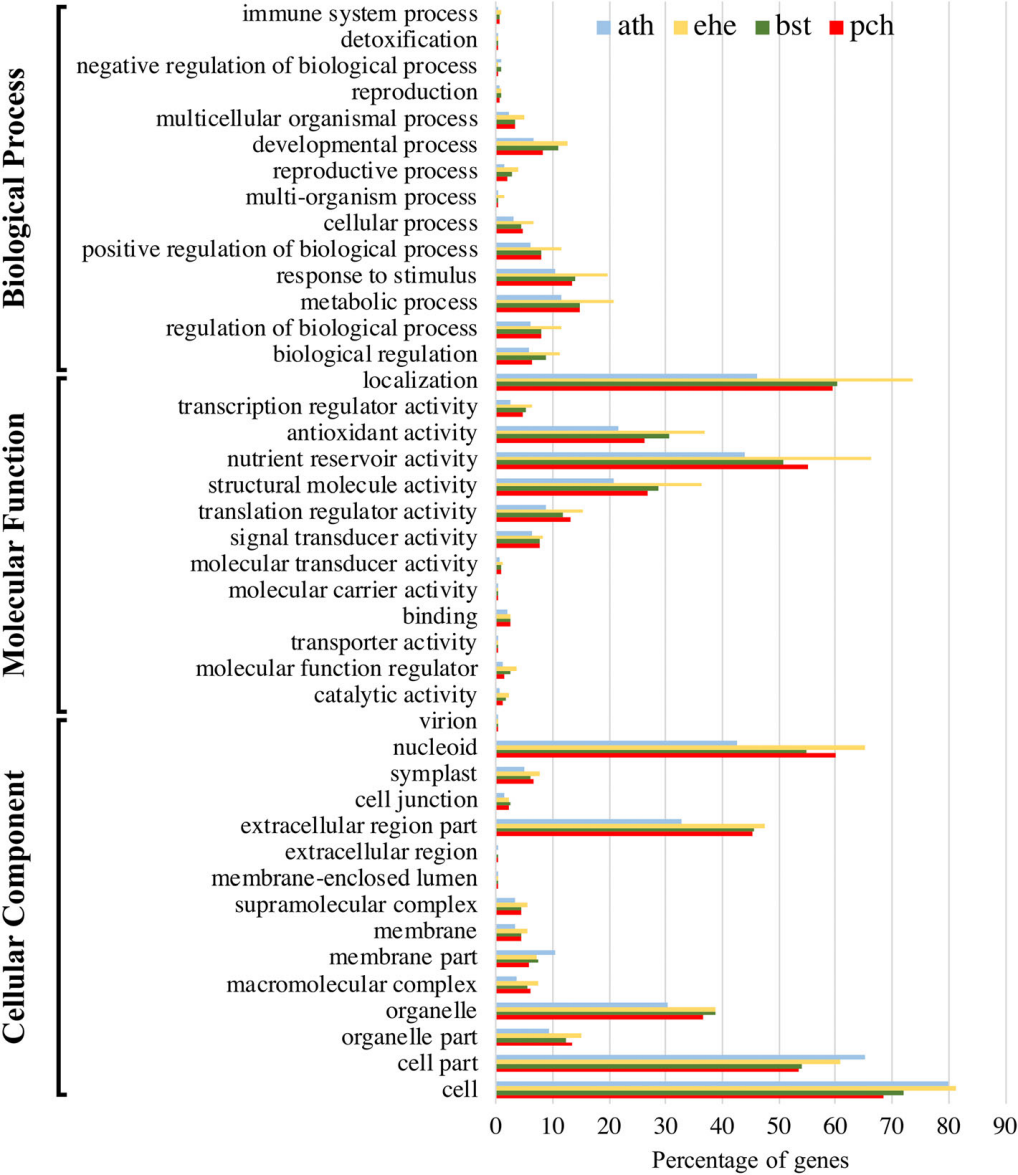
Gene Ontology (GO) annotation. Comparison of GO terms between *P. cheesemanii* (pch), *A. thaliana* (ath), *B. stricta* (bst) and *E. heterophyllum* (ehe)

Next, we used the *P. cheesemanii*, *B. stricta*, *E. heterophyllum* and *A. thaliana* genomes to analyze the GO enrichment patterns to further study the phylogenetic relationships of these species. The predicted gene annotations encompassed all major GO terms, suggesting that a core GO term set is present in the *P. cheesemanii* genome annotation (Fig. 2, Additional file 7). A comparison with the GO enrichment distributions of *B. stricta*, *E. heterophyllum* and *A. thaliana* revealed a similar pattern across all three GO categories in *P. cheesemanii* and *B. stricta*, while the *E. heterophyllum* pattern was considerably different from the other three species of Brassicaceae Lineage I (Fig. 2). Therefore, this result provides further support for the closer evolutionary grouping of *P. cheesemanii* with *B. stricta* of Brassicaceae Lineage I, than to *E. heterophyllum* of Lineage EII.

### Different UV-B responses in *Pachycladon cheesemanii* and *Arabidopsis thaliana*

The New Zealand environment is prone to high UV-B radiation levels naturally [30]. We therefore hypothesized that *P. cheesemanii* has evolved a higher UV-B radiation tolerance than its close relative, *A. thaliana*. Two accessions of *P. cheesemanii* were obtained from locations of relatively close proximity to each other. *P. cheesemanii* Kingston was collected just west of Kingston, New Zealand, at an altitude of ~ 500 m and *P. cheesemanii* Wye creek was collected 20 km north of Kingston at an altitude of ~ 300 m. The *P. cheesemanii* phenotypes were compared against those of the widely studied *A. thaliana* accession Col-0, which grows at an altitude of up to 100 m (www.arabidopsis.org), and the UV-B-resistant accession Kondara (distribution altitude: 1000–1100 m) [31, 32]. To test for responses to UV-B radiation, 28-day-old *A. thaliana* plants and 38-day-old *P. cheesemanii* plants, of similar plant size, were treated with UV-B radiation for 5 days to allow the manifestation of typical UV-B radiation phenotypic responses. A moderately high UV-B radiation (5.2 μmol m^− 2^ s^− 1^) was used to induce both UVR8-dependent and -independent responses.

**Fig. 3:**
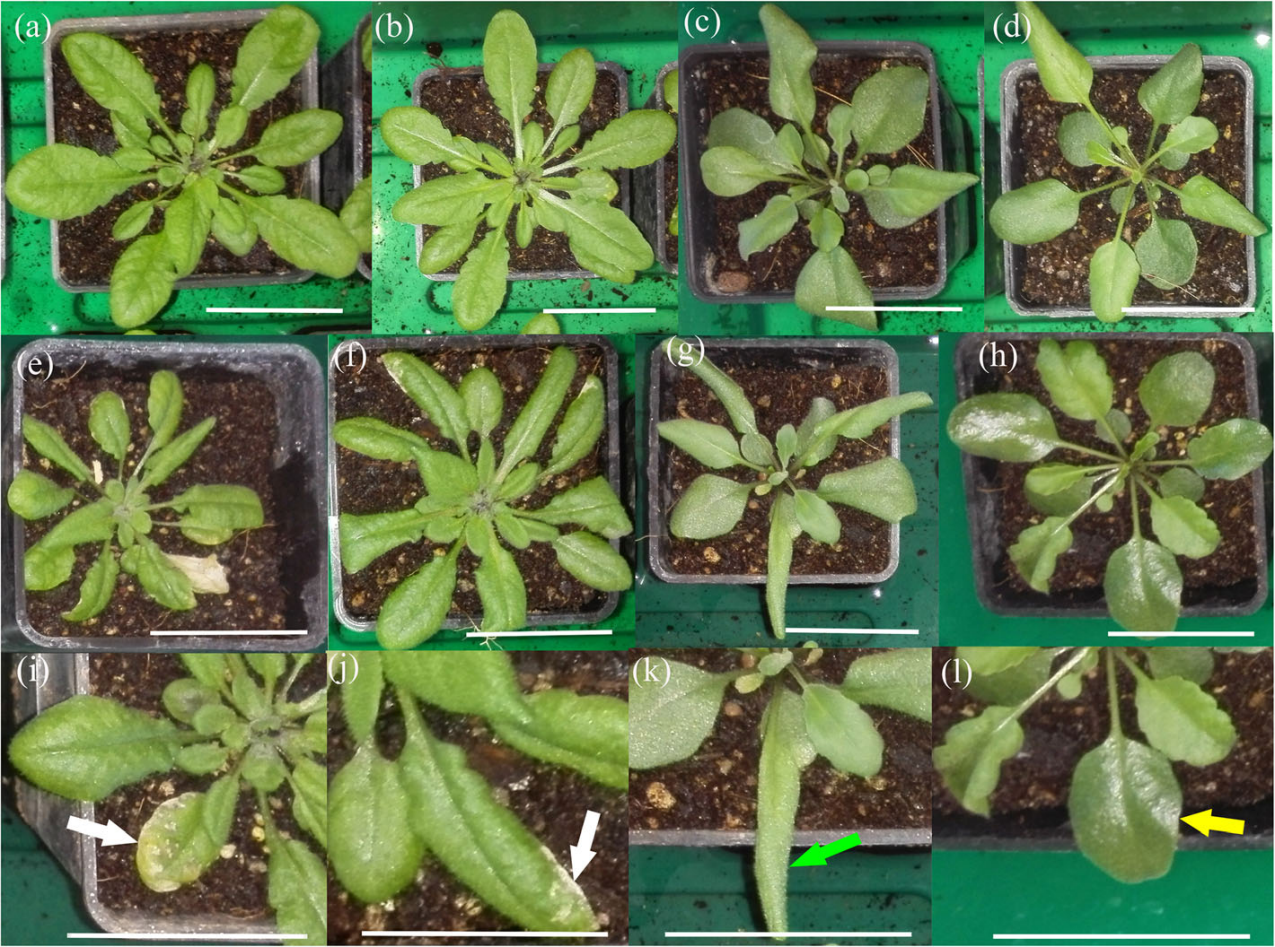
Twenty-eight-day-old *A. thaliana* and 38-day-old *P. cheesemanii* plants after a 5-day UV-B treatment. *A. thaliana* (28 days old) and *P. cheesemanii* (38 days old) plants were grown in long day conditions and subsequently transferred to UV-B-supplemented white light for 5 days (UV-B-5-day) or to white light only (control). **a** *A. thaliana* Col-0 **b** *A. thaliana* Kondara **c** *P. cheesemanii* Kingston **d** *P. cheesemanii* Wye creek plants grown under control conditions. **e** *A. thaliana* Col-0 **f** *A. thaliana* Kondara **g** *P. cheesemanii* Kingston **h** *P. cheesemanii* Wye creek plants after UV-B treatment. **i**-**l** Enlarged insets are shown for UV-B-treated plants (e-h) only. Arrows indicate necrotic lesions (white), leaf curling (green) and glossy appearance (yellow), respectively. Scale bars, 3.5 cm

**Fig. 4:**
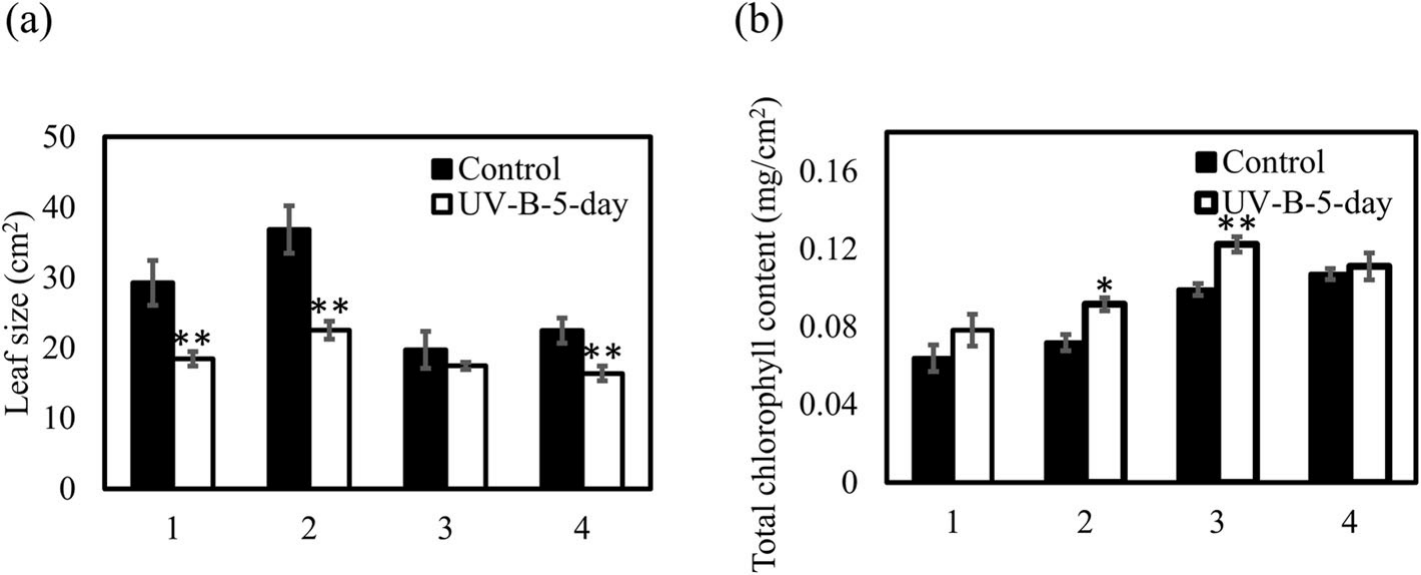
Total chlorophyll content and leaf size of *A. thaliana* and *P. cheesemanii* plants grown with and without UV-B radiation. *A. thaliana* (28 days old) and *P. cheesemanii* (38 days old) plants were grown in long day conditions and subsequently transferred to UV-B-supplemented white light for 5 days (UV-B-5-day) or to white light only (control). **a** Total leaf area. **b** Total leaf chlorophyll content. 1, *A. thaliana* Col-0; 2, *A. thaliana* Kondara; 3, *P. cheesemanii* Kingston; 4, *P. cheesemanii* Wye creek. Error bars represent SEM (Student’s *t*-test; *, *p* < 0.05; **, *p* < 0.01). Data were collected from 4 to 8 biological replicates

Leaves of UV-B radiation-treated *A. thaliana* Col-0 and Kondara plants were significantly smaller than leaves from untreated controls, and the Col-0 accession displayed more necrotic lesions on its leaves than Kondara (Fig. 3a, b, e, f, i, j and Fig. 4a). *P. cheesemanii* Wye creek plants showed a smaller but significant decrease in leaf size upon UV-B radiation compared to untreated controls. Interestingly, the leaf size of *P. cheesemanii* Kingston was not affected by UV-B radiation (Fig. 4a). All plants displayed some leaf curling and the leaves attained a glossy appearance, which was most apparent in *P. cheesemanii* Wye creek (Fig. 3c, d, g, h, k, l).

Next, we determined chlorophyll concentration in fully mature leaves of the different accessions. A significant increase in chlorophyll concentration was found in leaves of UV-B radiation-treated *A. thaliana* Kondara and *P. cheesemanii* Kingston plants, compared to untreated controls, while chlorophyll concentration did not change in *A. thaliana* Col-0 and *P. cheesemanii* Wye creek plants (Fig. 4b).

Taken together, our results support the notion that *P. cheesemanii* accessions exhibit a higher UV-B radiation tolerance than the *A. thaliana* accessions. Moreover, the two *P. cheesemanii* accessions responded to UV-B radiation in different ways.

### Distinct expression of UV-B radiation-inducible genes in *Pachycladon cheesemanii* and *Arabidopsis thaliana*

To further examine the UV-B radiation responses in *P. cheesemanii* and *A. thaliana*, we identified the *P. cheesemanii* homologues of 11 *A. thaliana* genes that function in the UVR8-dependent pathway and three homologues that play a role in the UVR8-independent pathway. The protein sequences of these genes were used to search the *P. cheesemanii* genome draft using TBLASTN. As a result, at least two potential copies of each gene were identified (Additional file 8 and Additional file 9), consistent with the polyploid nature of the *P. cheesemanii* genome. Primers for the *P. cheesemanii* genes were designed to amplify conserved protein-coding regions, such that both copies were expected to be amplified with equal efficiency.

**Fig. 5:**
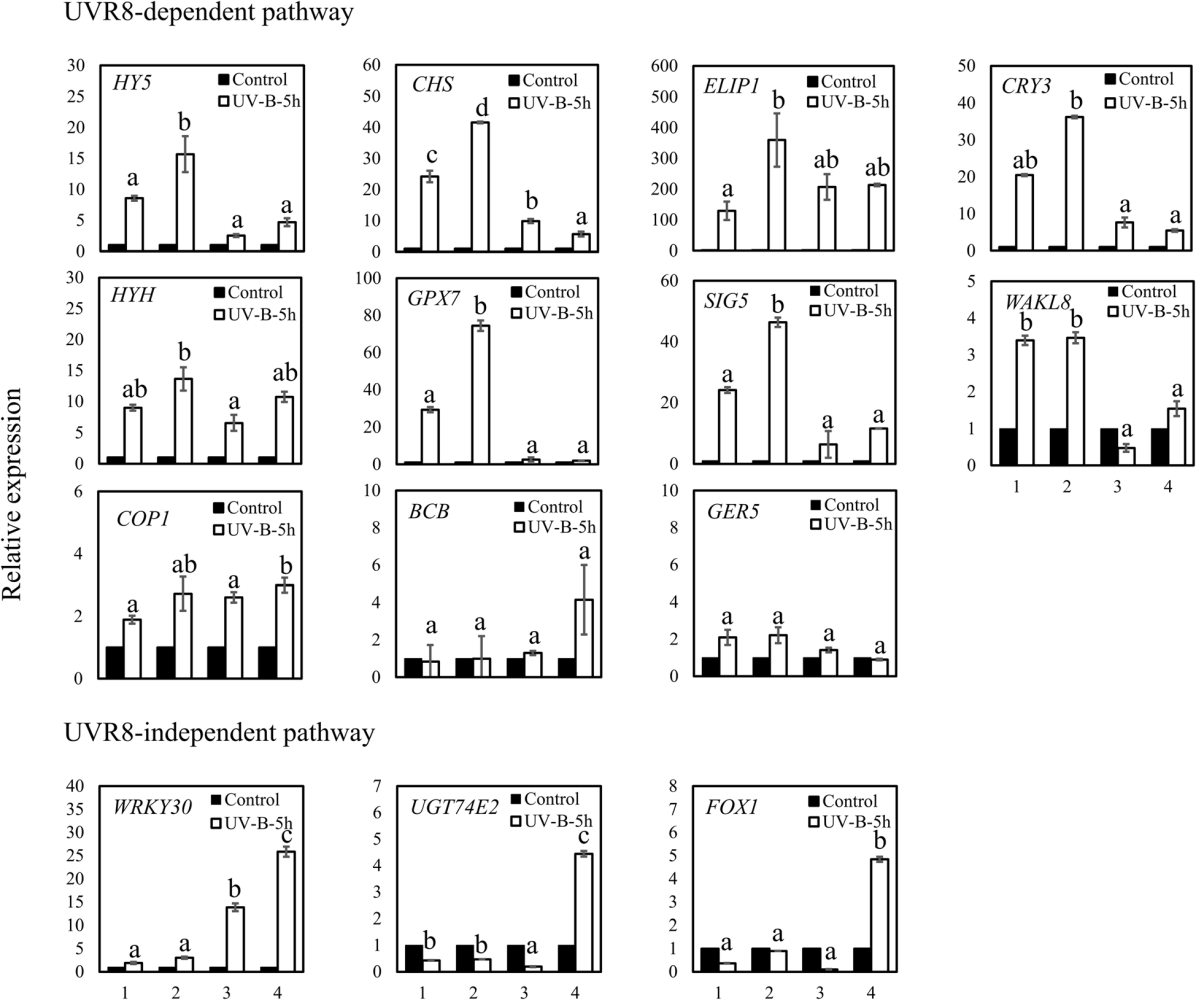
Relative expression of genes in UVR8-dependent and UVR8-independent UV-B response pathways in *A. thaliana* and *P. cheesemanii*. Six-week-old *A. thaliana* and nine-week-old *P. cheesemanii* plants were treated with white light (Control) or white light supplemented with UV-B radiation for 5 h (UV-B-5 h). Mature leaves were harvested for RT-qPCR analysis. The expression of transcripts indicated in each of the graphs was normalized to the reference genes of *A. thaliana* and *P. cheesemanii*, respectively. Relative expression (fold change) was calculated by dividing expression values of UV-B-treated plants with those of control-treated plants. 1, *A. thaliana* Col-0; 2, *A. thaliana* Kondara; 3, *P. cheesemanii* Kingston; 4, *P. cheesemanii* Wye creek. Data were from three independent biological replicates. Error bars represent SEM. Means with different lowercase letters indicate values of UV-B treated samples that are significantly different between the accessions analyzed (Tukey’s HSD, *p* < 0.05)

*P. cheesemanii* and *A. thaliana* plants were treated with UV-B radiation for 5 h to focus on early transcriptional effects and limit secondary responses. Gene expression of the selected genes was measured by quantitative real-time polymerase chain reaction (RT-qPCR). We initially measured 11 genes induced in *A. thaliana* by the UVR8-dependent pathway and found that eight (*HY5*, *HYH*, *CHS*, *ELIP1*, *CRYPTOCHROME 3* (*CRY3*), *GLUTATHIONE PEROXIDASE 7* (*GPX7*), *SIGMA FACTOR 5* (*SIG5*), and *WALL-ASSOCIATED RECEPTOR KINASE-LIKE 8* (*WAKL8*)) were upregulated by UV-B radiation in both *A. thaliana* accessions and three were not (*BCB*, a gene encoding a blue copper binding protein, *COP1*, and *GEM-RELATED 5* (*GER5*), which encodes a protein involved in hormone-mediated regulation of seed germination). Interestingly, while most of these genes were also upregulated in both *P. cheesemanii* accessions, the extent of upregulation was generally lower (Fig. 5).

We next quantified three genes of the UVR8-independent pathway, i.e., genes encoding *Arabidopsis thaliana* WRKY DNA-BINDING PROTEIN 30 (*WRKY30*), URIDINE DIPHOSPHATE GLYCOSYLTRANSFERASE 74E2 (*UGT74E2*), and FAD-LINKED OXIDOREDUCTASE (*FOX1*), and none of those was induced significantly in the *A. thaliana* accessions by 5.2 μmol m^− 2^ s^− 1^ of UV. However, the *WRKY30* homologue was upregulated in both *P. cheesemanii* accessions and the transcript levels of *UGT74E2* and *FOX1* were elevated in *P. cheesemanii* Wye creek, but not in *P. cheesemanii* Kingston. Thus, *A. thaliana* and *P. cheesemanii* accessions responded in different ways to UV-B radiation.

### Similar UV-B radiation-repair systems in *P. cheesemanii* and *A. thaliana*

**Fig. 6:**
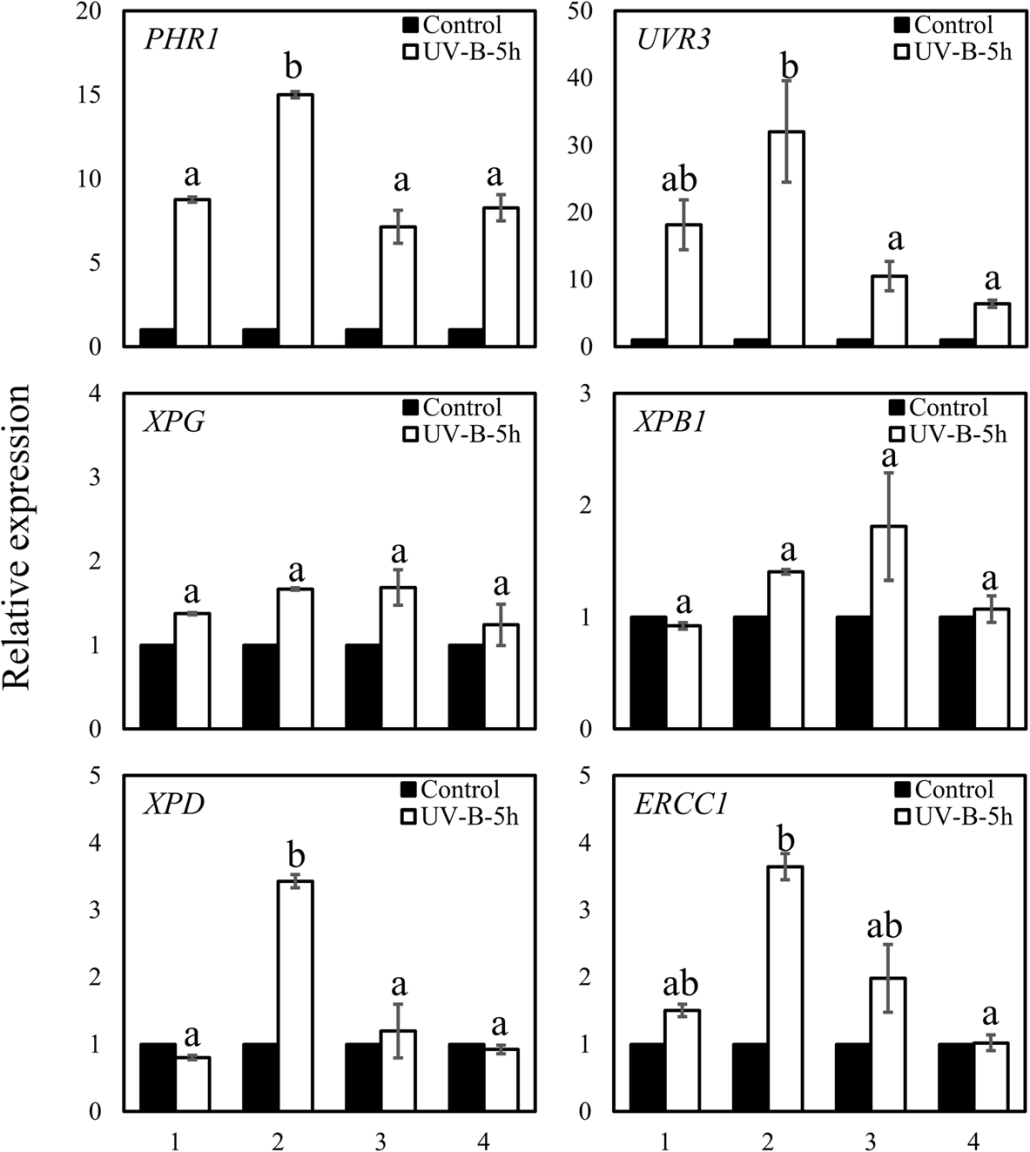
Relative expression of genes involved in DNA damage repair in *A. thaliana* and *P. cheesemanii*. Two genes involved in photorepair (*PHR1* and *UVR3*) and four genes involved in nucleotide excision repair were selected. Six-week-old *A. thaliana* and nine-week-old *P. cheesemanii* plants were treated with white light (Control) or white light supplemented with UV-B radiation for 5 h (UV-B-5 h). Mature leaves were harvested for RT-qPCR analysis. The expression of transcripts indicated in each of the graphs was normalized to the reference genes of *A. thaliana* and *P. cheesemanii*, respectively. Relative expression (fold change) was calculated by dividing expression values of UV-B-treated plants with thoes of control-treated plants. Data were from three independent biological replicates. 1, *A. thaliana* Col-0; 2, *A. thaliana* Kondara; 3, *P. cheesemanii* Kingston; 4, *P. cheesemanii* Wye creek. Error bars represent SEM. Means with different lowercase letters indicate values of UV-B treated samples that are significantly different between the accessions analyzed (Tukey’s HSD, *p* < 0.05)

Plants reduce susceptibility to UV radiation-induced damage through photorepair and dark repair systems [33]. Here, we identified *P. cheesemanii* homologues of six key genes involved in UV-B radiation-repair systems in *A. thaliana*. The UV-B radiation-induced transcript level of each gene was subsequently measured in *A. thaliana* and *P. cheesemanii* by RT-qPCR. In response to UV-B radiation, the two photorepair genes *PHOTOLYASE 1* (*PHR1*) and *UV REPAIR DEFECTIVE 3* (*UVR3*) were significantly upregulated in all four plant accessions, with *A. thaliana* Kondara showing the highest increase (Fig. 6). Interestingly, four genes involved in dark repair (nucleotide excision repair) or representing the *Arabidopsis* homologue of *Xeroderma Pigmentosum Complementation Group B1* (*XPB1*) from humans, i.e., *Arabidopsis thaliana XERODERMA PIGMENTOSUM GROUP D* (*XPD*), and a homologue of the human *ERCC1* gene (*ERCC1*), did not show obvious UV-B-induced transcript level changes in *A. thaliana* Col-0 or the two *P. cheesemanii* accessions, while *XPD* and *ERCC1* were upregulated 3.5-fold in *A. thaliana* Kondara (Fig. 6). Thus, the photorepair genes were upregulated in all plant accessions, while only *A. thaliana* Kondara showed an activated dark repair system.

## Discussion

### *Pachycladon cheesemanii* may originate from different Brassicaceae lineages

The two *Pachycladon* subgenomes have been proposed to result from a relatively young hybridization event, between unknown species, that occurred in New Zealand about 1.61 million years ago [5]. Here, we sequenced the *P. cheesemanii* genome and used it to help trace back the origin of the subgenomes of *Pachycladon* and to analyze UV-B radiation responses.

In our synteny analysis of 28 available Brassicaceae genomes, all eight species from tribes Boechereae and Camelineae (genera *Arabidopsis*, *Camelina*, *Capsella* and *Boechera*) displayed consistent high cumulative genome alignment percentages, regardless as to which of the eight species was combined with any of the 27 other species. Values for Boecherea (*B. stricta*) were the highest, suggesting that at least one of the *P. cheesemanii* subgenomes originated from the *Arabidopsis* lineage (Fig. 1a). Nevertheless, no cumulative values higher than 50% were found, suggesting that only one of the *Pachycladon* subgenomes has the same origin as these available Brassicaceae genomes. The findings are in general agreement with a recent Brassicaceae phylogenetic tree, established using 113 nuclear genes as markers, which proposes that *Pachycladon* is closely related to species of *Arabidopsis*, *Camelina*, *Capsella* and *Boechera* [29]. Similarly, Joly et al. [5] reported that one of the predicted ancestral subgenomes of *Pachycladon* has close phylogenetic relationships with *Boechera* and *Capsella* from the *Arabidopsis* lineage. However, the origin of the other subgenome is still unclear although phylogenetic analysis of the chloroplast *rbcL* gene in all Brassicaceae species suggested this genome to be the maternal ancestral subgenome [5]. Heenan et al. [34] proposed that the limited information on Brassicaceae species genomes hampered efforts to determine the origin of the second subgenome, and it may have originated from the tribe Smelowskieae or its close relatives that have subsequently gone extinct [34]. In contrast, we found that the second subgenome might have originated from Brassicaceae Lineage EII. The highest cumulative values were generated when using the combination of *B. stricta* and *E. heterophyllum*, the latter of which is from Brassicaceae Lineage EII [29]. The Eutrema tribe was predicted to be phylogenetically further away from *Pachycladon* than the Boechera tribe, however, it is close to the Brassiceae tribe phylogenetically, which could explain the *Brassica* trace in the *Pachycladon* genome found previously [5, 29]. Unfortunately, currently little genome information is available for Brassicaceae Lineage EII species, which limits the confident identification of the second *Pachycladon* subgenome ancestor. Altogether, we concluded that one of *Pachycladon* subgenomes has an origin similar to that of *B. stricta*, and that the other possibly arose from Brassicaceae Lineage EII.

### Distinct UV-B radiation tolerance responses in *A. thaliana* and *P. cheesemanii* species

Earlier research has reported that UV-B radiation-tolerant plants retained relatively high levels of chlorophyll after UV-B exposure [35]. Consistent with this, a higher chlorophyll concentration was found in UV-B radiation-tolerant *A. thaliana* Kondara as compared to the less UV-B radiation-tolerant accession *A. thaliana* Col-0 (Fig. 4b) [31]. The UV-B response is regulated by at least two pathways of which one is UVR8-dependent while the other does not require UVR8 [36]. The UVR8-dependent pathway induces the expression of six genes (*CHS*, *ELIP1*, *CRY3*, *GPX7*, *SIG5*, *WAKL8*) which is mediated by the transcription factors HY5 and HYH [26, 37]. Here we found that the genes encoding the transcription factors HY5 and HYH and the six above-mentioned genes were induced in response to the UV-B radiation, suggesting that the UVR8-dependent pathway was upregulated in both *A. thaliana* Kondara and Col-0 accessions. Of note, however, gene expression was generally more induced in *A. thaliana* Kondara than in Col-0 (Fig. 5). In addition, accession Kondara showed a greater induction of genes involved in photorepair and nucleotide excision repair (Fig. 6), which may allow it to recover from, or prevent UV-B-induced radiation stress [38]. Thus, the induction of chlorophyll biosynthesis genes and a stronger activation of HY5/HYH-pathway genes in Kondara than in Col-0, along with an increased DNA repair capacity in Kondara could be main contributors to its UV-B radiation tolerance.

Remarkably, leaf growth of the two *P. cheesemanii* accessions studied here was less affected by UV-B radiation than that of the *A. thaliana* accessions Kondara and Col-0; *P. cheesemanii* Kingston did not show a significant reduction of leaf area upon exposure to UV-B radiation compared to plants grown at control conditions (Fig. 4a). We found that the basal chlorophyll levels in the *P. cheesemanii* accessions were higher than those of the *A. thaliana* accessions. Furthermore, chlorophyll concentration in *P. cheesemanii* Kingston further elevated upon UV-B radiation and this correlated with its increased tolerance. Chlorophyll levels have been suggested to be an indicator of UV-B radiation tolerance [35] and the observed correlation between UV-B radiation tolerance, chlorophyll levels (Fig. 4b), and the ability to further increase chlorophyll levels upon UV-B radiation is consistent with that suggestion. The *P. cheesemanii* accessions generally induced genes of the UVR8-dependent pathway to a lower extent than the *A. thaliana* accessions, possibly as a result of a higher basal tolerance to UV-B radiation. Brown & Jenkins [26] identified three genes (*WRKY30*, *FOX1* and *UGT74E2*) in *A. thaliana* that were induced in response to 3–12 μmol m^− 2^ s^− 1^ UV-B radiation as part of the still poorly characterized UVR8-independent pathway. However, in contrast to the *A. thaliana* accessions, both *P. cheesemanii* accessions showed strong induction of the *WRKY30* gene at moderately high UV-B radiation levels of 5.2 μmol m^− 2^ s^− 1^. Crucially, only the Wye creek accession of *P. cheesemanii* induced expression of the two other genes (*FOX1* and *UGT74E2*), suggesting that the UVR8-independent pathway may be more important in *P. cheesemanii* than in *A. thaliana*. Thus, we found a positive correlation between chlorophyll content and UV-B radiation tolerance, and while the stronger induction of the UVR8-dependent pathway in *A. thaliana* accession Kondara may be at the basis of its UV-B radiation tolerance, the UVR8-independent pathway may play a more prominent role in *P. cheesemanii* accessions. Of further interest is that the lower UV-B radiation tolerance of the Wye creek accession correlated with a much stronger upregulation of genes involved in the UVR8-independent pathway, further supporting that this pathway plays an important role in coping with UV-B radiation damage in *P. cheesemanii*. Indeed, *P. cheesemanii* accessions may be particularly well suited for further exploring the UVR8-independent UV-B response pathway helping to increase our understanding of the genomic and genetic basis of plant adaptations to environmental UV-B stress.

## Conclusions

The polyploid nature of the *P. cheesemanii* genome may have contributed to its relatively high tolerance to UV-B radiation. We suggest that tolerance is achieved through the activation of the UVR8-independent response pathway. Our results are important for understanding the allopolyploidy nature of the *P. cheesemanii* genome and may be used to improve UV-B stress tolerance in Brassicaceae crop plants.

## Methods

### Plant growth and UV-B treatment

Seeds of *P. cheesemanii* Kingston (geographical coordinates in decimal degrees − 45.3273, 168.7078) and Wye creek (geographical coordinates in decimal degrees − 45.1398, 168.7672) were provided by Dr. Claudia Voelckel (Max Planck Institute for Chemical Ecology, Jena, Germany) and Dr. Peter Heenan (Wildland Consultants Ltd., Rotorua, New Zealand). Seeds of *Arabidopsis thaliana* (L.) Heynh. accessions Col-0 and Kondara were obtained from the *Arabidopsis* Biological Resource Center (*ABRC*; https://abrc.osu.edu). Seeds of both species were sown and germinated in wet Seed Raising Mix® soil from Daltons (Matamata, New Zealand) and seedlings were grown under a 16-h light (200 μmol m^− 2^ s^− 1^ cool-white fluorescent tube)/8-h dark (long-day) regime at 22 °C. For UV-B treatment, the two *P. cheesemanii* accessions were sown 10 days earlier than the *A. thaliana* plants to achieve the same plant size. After 28 (*A. thaliana*) or 38 (*P. cheesemanii*) days of growth, the plants were transferred to the UV-B radiation chamber where they were subjected to normal white light (200 μmol m^− 2^ s^− 1^ cool-white fluorescent tube) supplemented with 5.2 μmol m^− 2^ s^− 1^ UV-B (290–320 nm) for UV-B treatments, while the control plants were kept under white light conditions. The UV-B fluorescent tubes used in the chamber were Q-Panel 313 (Q-Lab Corp, Cleveland, OH, USA), which were wrapped in 0.13-mm-thick cellulose diacetate foil (Clarifoil; Courtaulds Ltd., Derby, UK) to remove wavelengths < 290 nm. The chamber was split into a UV-B+ zone and a UV-B− zone separated by a central curtain of UV-B opaque film (Lumivar; BPI Visqueen, Ardeer, UK). For the UV-B− zone, the UV-B tubes were wrapped in the same UV-B opaque film [39]. UV-B treatments were quantified at plant canopy height with an Optronics OL-756 UV-VIS Spectroradiometer (Optronic Laboratories, Gooch and Housego, FL, USA) equipped with integrating sphere. Spectroradiometric scans of the controlled environment chamber confirmed that the biologically effective UV dose was < 0.01 kJ m^− 2^ d^− 1^ in the UV-B− zone. UV-B-treated and nontreated plants were collected 5 days after the treatment. For quantification of transcript abundance, seedlings were grown under a 10-h light (200 μmol m^− 2^ s^− 1^ cool-white fluorescent lamp)/14-h dark (short-day) regime at 22 °C for 6 weeks (*A. thaliana*) and 9 weeks (*P. cheesemanii*) to obtain a large leaf area to maximise UV-B radiation absorption. The plants were subsequently mock treated or UV-B irradiated for 5 hours, after which leaf tissue was collected.

### Library preparation and Illumina sequencing

Genomic DNA was prepared from *P. cheesemanii* Kingston leaves. DNA was extracted using a modified CTAB DNA extraction protocol [40] with extra steps to remove chloroplast DNA. Purified *P. cheesemanii* genomic DNA was used to generate 550-bp paired-end (PE) and 1.5-kb to 15-kb mate-pair (MP) sequencing libraries. Following library quality control, the libraries were sequenced on two lanes of an Illumina HiSeq 2500 machine, with 2 × 125 PE and 2 × 125 MP reads generating a total of ~ 56 Gb raw sequencing data. This represents a theoretical coverage of 100-fold based on the estimated size of the *P. cheesemanii* genome (Additional file 10). PE and MP library construction and Illumina sequencing were performed by New Zealand Genomics Limited (NZGL, Otago, New Zealand).

### Genome assembly and assessment

To assess data quality, raw reads were analyzed using SolexaQA++ v3.1.7 [41] (Cox et al., 2010). Adaptors were trimmed using trim_galore v0.4.1 [42], and the processed reads were used for assembly. SOAPdenovo [43] and Platanus v1.2.4 [44] were used for assembly and different k-mer sizes were used to produce multiple assemblies for further quality assessment (Additional file 2). Assembly statistics were generated by QUAST v4.1 and Bowtie 2 [45, 46]. Leaf transcriptome data (full transcripts) of *P. cheesemanii* [6] (downloaded from NCBI) were used for mapping to the genome assemblies using PASA v2.0.2 [47]. BUSCO v3.0.2 [48] (dataset: “embryophyta_odb9” containing 1440 orthogroups, downloaded from http://busco.ezlab.org) was used to evaluate the completeness of the assemblies. The result metrics from the tools mentioned above were used to select the best assembly (Additional file 2). Samtools [49] was used for identifying SNPs. After quality filtering, the approximate genome size was determined using the total base number of sequencing data and sequencing depth being derived from the k-mer distribution using the formula [50]: G = (N×(L-K + 1)-B)/D; G, genome size; N, number of reads; L, length of reads; K, length of k-mer; B, low-frequency k-mers with occurrence less than four times; D, sequencing depth corresponding to selected k-mer.

### Identification of repeats

RepeatModeler v1.0.8 [51], employing RepeatScout, Tandem Repeats Finder and RECON modules, was used to construct a *de novo* repeats library for *P. cheesemanii*. LTR_finder v1.0.5 [52], TransposonPSI v08222010 [53], and MITE-hunter v11–2011 [54] were used to identify Long Terminal Repeats (LTRs), retrotransposons, and Miniature Inverted repeat Transposable Elements (MITEs), respectively. Viridiplantae repeats were extracted from Repbase repeat libraries [55]. The assembly was masked by *de novo* and Viridiplantae repeats using RepeatMasker v4.0.5 [56] to estimate the non-redundant genomic repeat content. The repeats were further classified using the RepeatClassifier module of RepeatModeler.

### Functional annotation

A combination of *ab initio* and homology-based annotation methods was used in the Maker pipeline v2.31.8 [57] for gene prediction. Briefly, AUGUSTUS v3.2.2 [58] was used with an *Arabidopsis* training set and leaf transcriptome alignments from PASA were used to improve and provide evidence for gene prediction. Gene annotation was done using BLASTP v2.6.0 [59] and BLASTX against Uniprot (Swissprot + TrEMBL) and the NCBI nr database (only BLASTX), restricting the search to Viridiplantae using taxonomy filter, best hit, E-value cutoff of 1e-20, query coverage of ≥50% and percentage identity ≥50%. Annotations for each gene were selected as per the following order of preferences: 1) Swissprot (BLASTP), 2) Swissprot (BLASTX), 3) Uniprot (BLASTP), 4) Uniprot (BLASTX), 5) NCBI-nr (BLASTX). InterProScan v5.22–61.0 [60] was used for function annotation of the protein domains and families using all available databases in InterProScan. GO annotations were obtained from Uniprot database. Transcription factors were predicted using PlantTFDB v4.0 [61]. KAAS [62] was used to obtain KEGG pathway annotations. MISA [63] was used to predict SSR markers. Infernal v1.1.2 [64] was used to predict non-coding RNAs with an E-value cutoff of 1e-5 and removing low-scoring overlaps.

### Comparative genomics

Genomes of available Brassicaceae species were downloaded from NCBI and Phytozome [65]. Nucmer from MUMmer v3.23 [66] was used to align *P. cheesemanii* genome sequences against each Brassicaceae genome to perform synteny analysis. Delta filter was applied to select only one-to-one alignments. OrthoFinder v2.1.2 [67] was used to find orthologs among the protein sequences of the selected genomes. GO annotations for *A. thaliana* were obtained from Araport11 [68], and for *B. stricta* and *C. sativa*, the GO annotations were obtained in the same way as for *P. cheesemanii*. The dendrogram was plotted using iTOL [69]. Overlaps between orthogroups were plotted using the ClusterVenn utility in OrthoVenn [70].

### Chlorophyll content measurement

Chlorophyll content was measured by using a modified chlorophyll analysis protocol based on Ignat et al. [71]. Two fresh mature leaves were immersed in 10 ml 95% ethanol, and tubes were placed in a cool room (4 °C) for 24 h. The chlorophyll-extraction solution was then collected into new tubes, and a further 10 ml ethanol was added to the leaves for another 4 h to collect the remainder of the chlorophyll. The combined 20 ml chlorophyll-extraction solution was mixed well and 2 ml was used to quantify the chlorophyll content at 664 and 649 nm by using the following formulas [71]:

$$ \mathrm{Chlorophyll}\ a=\left(13.36\times A664-5.19\times A649\right) $$

$$ \mathrm{Chlorophyll}\ b=\left(27.43\times A649-8.12\times A664\right) $$

$$ \mathrm{Total}\ \mathrm{chlorophyll}=\left(5.24\times A664+22.24\times A649\right) $$

Subsequently, chlorophyll contents were normalized to the corresponding total leaf areas that were used to extract chlorophyll.

### RNA extraction, cDNA synthesis and RT-qPCR

Total RNA was extracted from mature leaves with a Quick-RNA MiniPrep Kit (Zymo Research, CA, USA) and treated with DNase I to remove genomic DNA contamination. Reverse transcription was performed with an oligo (dT) primer using a Transcriptor First Strand cDNA Synthesis Kit (Roche, Basel, Switzerland). The gene sequences and primers used in RT-qPCR are listed in Additional files 9 and 11. RT-qPCR was performed using a LightCycler 480 SYBR Green I Master kit (Roche) (LightCycler 480; Roche). For real-time PCR quantification, *AT4G29130* was used as a reference gene in *A. thaliana*, and the homologue of *AT4G34270* was used as a reference gene in *P. cheesemanii* based on previous reports [72, 73]. The *P. cheesemanii* homologue of *AT4G34270* was identified by applying TBLASTN.

## Supplementary information

**Additional file 1.** K-mer analysis for estimating the genome size of *P. cheesemanii*. The genome size was estimated by using the formula: G = (N x(L-K + 1)-B)/D. G, genome size; N, number of reads; L, length of reads; K, length of k-mer; B, low-frequency k-mers with occurrence less than four times; D, coverage depth corresponding to selected k-mer. 41,51,61,71,81,91, and 101-mer sizes were analyzed, and the coverage depth of 41-mer was selected for genome size estimation.

**Additional file 2.** Statistics of different assemblies for *Pachycladon cheesemanii*.

**Additional file 3.** Repeats content estimation and classification for *Pachycladon cheesemanii*.

**Additional file 4.** Characteristics of the 20 longest scaffolds of the *P. cheesemanii* genome assembly. 1) SNPs, 2) GC content (bin: 5 kb; axis: 0–50%), 3) repeats, 4) read coverage (bin: 5 kb), 5) predicted genes, 6) PASA alignments against the leaf transcriptome, 7) distribution of gaps (Ns).

**Additional file 5.** Distribution of Simple Sequence Repeats (SSR) markers identified using MISA.

**Additional file 6.** KEGG pathway annotation for all genes obtained from KEGG Automated Annotation Server (KAAS).

**Additional file 7.** Gene Ontology annotation for all genes obtained from BLAST similarity searches.

**Additional file 8.**Comparison of transcript and genomic DNA sequences between *A. thaliana* *CHS* and two *P. cheesemanii* homologues. The two *P. cheesemanii* homologues show obvious sequence differences from *A. thaliana*, and slight differences between them. Full sequences of the *P. cheesemanii* genes are given in Table S3. The indicated sequences were aligned using CLUSTAL OMEGA (1.2.4) (https://www.ebi.ac.uk/Tools/msa/clustalo/) and the conserved nucleotide sequences are indicated by asterisks below the sequences.

**Additional file 9.** Sequences of genes used for RT-qPCR analysis.

**Additional file 10.** Read statistics of libraries used for sequencing.

**Additional file 11.** List of primers used for RT-qPCR analysis.

## Data Availability

Sequencing data are available from the NCBI Bioproject database (www.ncbi.nlm.nih.gov/bioproject) under ID: PRJNA475190.

## Abbreviations

ABRC: Arabidopsis Biological Resource Center
BCB: Blue Copper Binding protein
CHS: Chalcone Synthase
COP1: Constitutive Photomorphogenic 1
CRY3: Cryptochrome 3
EII: Expanded Lineage II
ELIP1: Early Light-Inducible Protein 1
ERCC1: Excision Repair 1
F3H: Flavanone 3-Hydroxylase
FLS1: Flavonol Synthase 1
FOX1: FAD-linked Oxidoreductase 1
GER5: Gem-Related 5
GPX7: Glutathione Peroxidase 7
HY5: Elongated Hypocotyl 5
HYH: HY5-Homolog
KAAS: KEGG Automated Annotation Server
LTRs: Long Terminal Repeats
MITEs: Miniature Inverted repeat Transposable Elements
MP: Mate-Pair
MUMs: Maximal Unique Matches
NCBI: National Center for Biotechnology Information
NZGL: New Zealand Genomics Limited
PE: Paired-End
PHR1: Photolyase 1
RT-qPCR: Quantitative Real-Time Polymerase Chain Reaction
SIG5: Sigma Factor 5
SSR: Simple Sequence Repeat
TF: Transcription Factor
UGT74E2: Uridine Diphosphate Glycosyltransferase 74E2
UV-A: Ultraviolet A
UV-B: Ultraviolet B
UV-C: Ultraviolet C
UVR3: UV Repair defective 3
UVR8: UVB-Resistance 8
WAKL8: Wall-Associated receptor Kinase-Like 8
WRKY30: WRKY DNA-binding protein 30
XPB1: Xeroderma Pigmentosum complementation group B1
XPD: Xeroderma Pigmentosum group D
