# Supplementary material for: Genome draft of the Arabidopsis relative Pachycladon cheesemanii reveals novel strategies to tolerate New Zealand’s high ultraviolet B radiation environment
Source: BMC Genomics. 2019 Nov 12;20:838. doi: 10.1186/s12864-019-6084-4 (PMC6849220; doi:10.1186/s12864-019-6084-4)
Supplement: Supplementary file 1 — Additional file 1. K-mer analysis for estimating the genome size of P. cheesemanii. The genome size was estimated by using the formula: G = (N x(L-K + 1)-B)/D. G, genome size; N, number of reads; L, length of reads; K, length of k-mer; B, low-frequency k-mers with occurrence less than four times; D, coverage depth corresponding to selected k-mer. 41,51,61,71,81,91, and 101-mer sizes were analyzed, and the coverage depth of 41-mer was selected for genome size estimation. [file 12864_2019_6084_MOESM1_ESM.docx]

**Additional file 1.** **K-mer analysis for estimating the genome size of *P. cheesemanii*.** The genome size was estimated by using the formula: G=(N x(L-K+1)-B)/D. G, genome size; N, number of reads; L, length of reads; K, length of k-mer; B, low-frequency k-mers with occurrence less than four times; D, coverage depth corresponding to selected k-mer. 41,51,61,71,81,91, and 101-mer sizes were analyzed, and the coverage depth of 41-mer was selected for genome size estimation.
