# Supplementary material for: Genome draft of the Arabidopsis relative Pachycladon cheesemanii reveals novel strategies to tolerate New Zealand’s high ultraviolet B radiation environment
Source: BMC Genomics. 2019 Nov 12;20:838. doi: 10.1186/s12864-019-6084-4 (PMC6849220; doi:10.1186/s12864-019-6084-4)
Supplement: Supplementary file 4 — Additional file 4. Characteristics of the 20 longest scaffolds of the P. cheesemanii genome assembly. 1) SNPs, 2) GC content (bin: 5 kb; axis: 0–50%), 3) repeats, 4) read coverage (bin: 5 kb), 5) predicted genes, 6) PASA alignments against the leaf transcriptome, 7) distribution of gaps (Ns). [file 12864_2019_6084_MOESM4_ESM.docx]

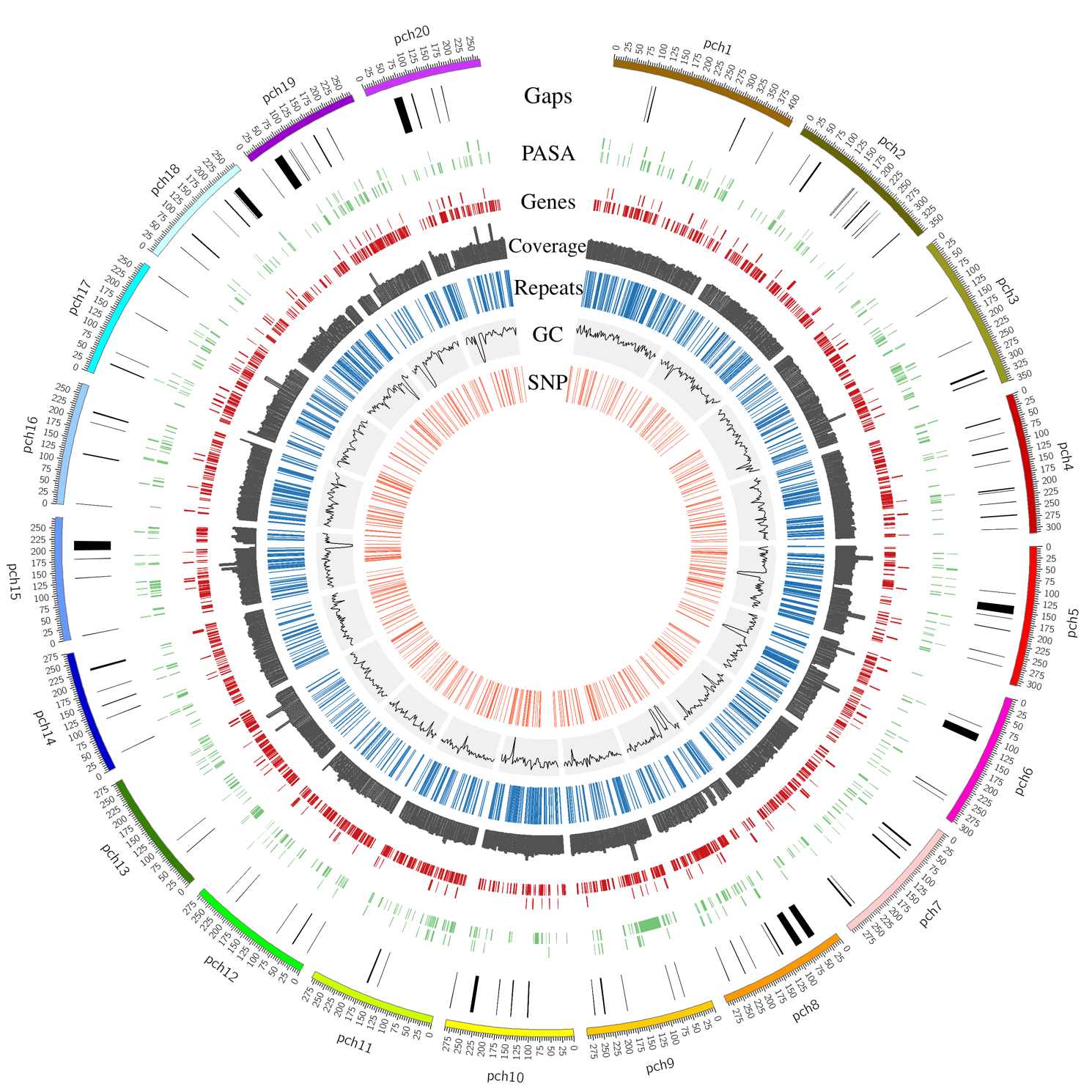


**Additional file 4. Characteristics of the 20 longest scaffolds of the *P. cheesemanii* genome assembly.** 1) SNPs, 2) GC content (bin: 5 kb; axis: 0 – 50%), 3) repeats, 4) read coverage (bin: 5 kb), 5) predicted genes, 6) PASA alignments against the leaf transcriptome, 7) distribution of gaps (Ns).
