# Supplementary material for: Genome draft of the Arabidopsis relative Pachycladon cheesemanii reveals novel strategies to tolerate New Zealand’s high ultraviolet B radiation environment
Source: BMC Genomics. 2019 Nov 12;20:838. doi: 10.1186/s12864-019-6084-4 (PMC6849220; doi:10.1186/s12864-019-6084-4)
Supplement: Supplementary file 8 — Additional file 8.Comparison of transcript and genomic DNA sequences between A. thaliana CHS and two P. cheesemanii homologues. The two P. cheesemanii homologues show obvious sequence differences from A. thaliana, and slight differences between them. Full sequences of the P. cheesemanii genes are given in Table S3. The indicated sequences were aligned using CLUSTAL OMEGA (1.2.4) (https://www.ebi.ac.uk/Tools/msa/clustalo/) and the conserved nucleotide sequences are indicated by asterisks below the sequences. [file 12864_2019_6084_MOESM8_ESM.docx]

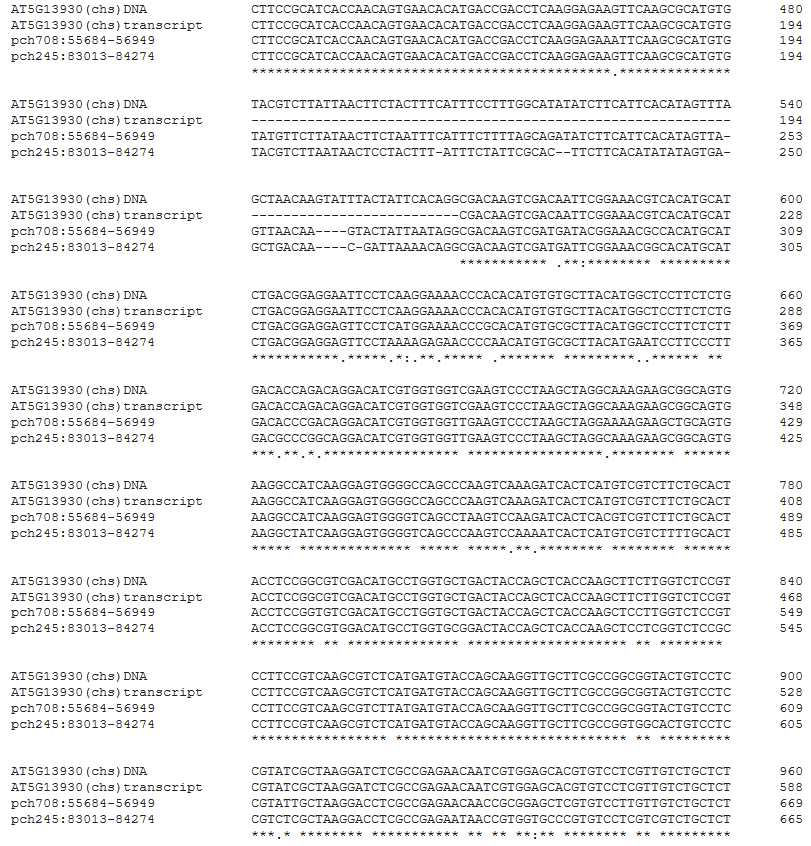


**Additional file 8. Comparison of transcript and genomic** **DNA sequences between *A. thaliana CHS* and two *P. cheesemanii* homologs.** The two *P. cheesemanii* homologs show obvious sequence differences from *A. thaliana*, and slight differences between them. Full sequences of the *P. cheesemanii* genes are given in Table S3. The indicated sequences were aligned using CLUSTAL OMEGA (1.2.4) (https://www.ebi.ac.uk/Tools/msa/clustalo/) and the conserved nucleotide sequences are indicated by asterisks below the sequences.
